# Antidiabetic, anti-inflammatory, antioxidant, and cytotoxicity potentials of green-synthesized zinc oxide nanoparticles using the aqueous extract of *Helichrysum cymosum*

**DOI:** 10.1007/s13205-024-04125-0

**Published:** 2024-11-04

**Authors:** Achasih Q. Nkemzi, Kunle Okaiyeto, Omolola Oyenihi, Chinyerum S. Opuwari, Okobi E. Ekpo, Oluwafemi O. Oguntibeju

**Affiliations:** 1 Phytomedicine and Phytochemistry Group, Department of Biomedical Sciences, Faculty of Health and Wellness Sciences, Cape Peninsula University of Technology, Bellville, 7535 South Africa; 2 Department of Medical Bioscience, University of the Western Cape, Bellville, Cape Town, 7530 South Africa; 3 Department of Biological Sciences, College of Medicine and Health Sciences, Khalifa University, P. O. Box 127788, Abu Dhabi, United Arab Emirates

**Keywords:** Alpha-glucosidase, Alpha-amylase, Hexagonal structure, C3A hepatocyte, L6 myocyte, Characterization

## Abstract

The current research involved the synthesis of zinc oxide nanoparticles (ZnO-NPs) using an aqueous extract of *Helichrysum cymosum* shoots, and subsequent characterization via different analytical methods, such as UV–Vis spectroscopy, Scanning electron microscope (SEM), Energy-dispersive X-ray spectroscopy (EDX), X-ray diffraction (XRD), Transmission electron microscope (TEM), and zeta potential. The biological effects of the ZnO-NPs were then tested against C3A hepatocyte cells and L6 myocyte cell lines via series of analysis, including cytotoxicity, antioxidant, anti-inflammatory, and antidiabetic effect via enzymatic inhibition. The UV–Vis analysis showed a maximum absorption spectrum at 360, and the TEM analysis reveals a spherical and hexagonal structures, with an average dimension of 28.05–58.3 nm, and the XRD reveals a crystalline hexagonal structure. The zeta potential evaluation indicated that the ZnO-NPs are relatively stable at − 20 mV, and the FTIR analysis identified some important functional group associated with phenolics, carboxylic acid, and amides that are responsible for reducing and stabilizing the ZnO-NPs. The synthesized ZnO-NPs demonstrated cytotoxic effects on the cell lines at higher concentrations (125 µg/mL and 250 µg/mL), complicating the interpretation of the results of the inflammatory and antioxidant assays. However, there was a significant (*p* < 0.05) increase in the inhibitions of pancreatic lipase, alpha-glucosidase, and alpha-amylase, indicating beneficial antidiabetic effects.

## Introduction

Nanotechnology applications span a wide range of scientific fields, including agriculture, cosmetics, the food industry, material science, engineering, medical science, etc. (Akintelu and Folorunso 2020; Paul et al. 2020; Singh et al. 2021; Okaiyeto et al. 2021). The unique dimension (1–100 nm) and easy permeability of nanoparticles across biological barriers have attracted much attention (Abel et al. 2021; Falih et al. 2022; Singh et al. 2021). The ultrafine particle size of nanoparticles offers range of benefits, such as improved compound solubility, lower medicinal doses, and enhanced absorption, making them an attractive option for drug formulation and delivery (Ikbal et al. 2023). Nanoparticles have exhibited impressive potentials as multifunctional agent, merging diagnostics and therapeutic functions into a single platform for targeting drug delivery, environmental-responsive drug release, molecular imaging, and enhanced treatment outcome (Jia et al. 2020).

Several approaches have been used to synthesize nanoparticles, including chemical, physical, and biological methods (Varadharaj et al. 2020; Brayami et al. 2020; Donga and Chanda 2022). However, the use of chemical and physical methods has become unattractive, because it is time-consuming, complicated, costly, and often associated with toxicity and limited biocompatibility (Donga and Chanda 2022). Therefore, alternative biosynthesis routes using plant extracts have recently gained attention as they pose little or no environmental hazard. This “green” method is preferable, because its protocols are simple, affordable, clean, and involve the use of eco-friendly solvents for extraction; thus, this method can be considered appropriate for large-scale production of nanoparticles (Ochieng et al. 2015; Brayami et al. 2020; Aldeen et al. 2022; Govindan et al. 2020). Important biological molecules present in the different parts of a plant, e.g., alkaloids, amino acids, flavonoids, and proteins, are known to play a vital role during green synthesis by reducing metal ions to metal nanoparticles and stabilising the synthesized nanoparticles (Ochieng et al. 2015; Aldeen et al. 2022).

Nanoparticles synthesized from metal and metal oxides, such as gold (Au), silver (Ag), selenium, copper (Cu), copper (II) oxide (CuO), zinc oxide (ZnO), platinum (Pt), and palladium (Pd), have presented great potential when applied in different domains (Donga and Chanda 2022; Gebre 2023). Zinc oxide nanoparticles (ZnO-NPs) have gained significant attention among other metal nanoparticles due to their specific physical and chemical properties (Jiang et al. 2018; Issam et al. 2021) such as semiconducting properties with a band gap of 3.37 eV and a high excitation energy of 60 meV. Other notable properties include biological, chemical, electrical, physical, environmental, biocompatibility, low cost, and non-toxic properties (Thema et al. 2015; Kumar et al. 2018; Abel et al. 2021; Donga and Chanda 2022). In addition, ZnO nanoparticles synthesized from different parts of plants have been reported with diverse biological activities, such as antimicrobial, anti-inflammatory, antioxidant, cytotoxicity, antidiabetic, anticancer, and photocatalytic properties (Saratale et al. 2018; Govindan et al. 2020; Ifeanyichukwu et al. 2020; Ahmed 2022; Donga and Chanda 2022). ZnO is predominately used in metal nanoparticle surveys (Ifeanyichukwu et al. 2020), and the green biosynthesis approach (ZnO) has been reported to be a promising option for producing ZnO-NPs. Metal-oxide NPs have been explored as potential therapeutic agents for ailments such as diabetes (Thema et al. 2015; Mishra et al. 2017).

Diabetes mellitus (DM) is a complex and chronic metabolic disorder emanating from increased blood sugar levels (hyperglycemia) (Ashrafizadeh et al. 2020). It results from impaired glucose metabolism due to abnormal insulin secretion, action, or both (Dhas et al. 2016; Brayami et al. 2020). Prolonged DM has been associated with severe health complications, such as nephropathy, neuropathy, retinopathy, and liver damage which, if left unattended to, might result in severe morbidity and mortality (Virk 2017). An increase in DM worldwide has been associated with ageing, lifestyle, and urbanization. Global epidemiological data indicate a significant rise in the number of individuals with DM over the past three decades, increasing from 160 to 410 million. The prevalence rate is estimated at 420 million individuals in 2019, and is projected to reach 690 million by 2040 (Jayarambabu et al. 2020). The management of DM has been a serious public health concern and requires strategic interventions (Badeggi et al. 2020). Several efforts in managing DM have been reported, such as lifestyle and nutritional changes and pharmacological intervention. An alternative way of managing DM is the use of herbal medicine, which has shown promising results and possesses hypoglycemic potential (Virk 2018).

Zn is a trace element that plays a critical enzymatic and cellular role in the human body, such as immune functions, apoptosis, metabolic regulations, oxidative equilibrium, and metabolic and signal transduction. Zinc metal has been reported to ameliorate diabetic complications such as nephropathy and cardiomyopathy by enhancing the mechanisms of insulin signalling pathways (Tang 2019). Additionally, the role of zinc as a cofactor to over 300 enzymes underscores its importance in maintaining cellular homeostasis influencing protein production, cell cycle progression and regulation, and DNA replication and apoptosis (Hamed et al. 2023). Owing to the importance of zinc to the human body and the quest for alternative medicine to treat DM, ZnO-NPs have been the preferred option used to deliver zinc in many disease therapies because of the beneficial role played in numerous enzymatic and cellular activities of the body (Ahmed 2022). The role played by ZnO-NPs in reducing mRNA inflammatory cytokines through the inhibition of activation of NF-kB has also been documented (Paul et al. 2020).

Several ethnopharmacological studies have revealed the beneficial use of plant-mediated medicines in treating different ailments, since plant-based medicinal options are known to be affordable, reliable, and less toxic (Ansaril et al. 2022, Sagbo and Hussein 2022). Furthermore, the emergence of plant-based mediated nanoparticle research has increased in recent years due to beneficial biomolecules, such as alkaloids, flavonoids, glucosides phenolic, and protein they possess, which are responsible for the reduction of metal ions to nanoparticles (Okaiyeto et al. 2021). The important role played by these plant phytochemical constituents has prompted the search for novel antidiabetic and anti-inflammatory drugs with minimal toxicity. ZnO-NP offers a potential therapeautic option for DM and its associated complications (Ikbal et al. 2023; Sharmaet al. 2023; Al Aamri et al. 2024). *H. cymosum* belongs to the Asteraceae family and has been reported to possess notable biomolecules, such as flavone, 5-hydroxy-8 methoxy-7-prenyloxyflavonone helihumolone, helichromachalcone and phloroglucinol derivatives, sesquiterpenes, and chalcones (Van Vuuren et al. 2006; Lourens et al. 2011; Heyman 2013; Jadalla et al. 2022). These biomolecules have been tested in different biomedical applications, such as antioxidant, anti-inflammatory, antifungal, antiviral, antimicrobial, antidiabetic, and cytotoxicity activities from these species (Matanzima 2014; Jadalla et al. 2022; Maroyi 2019). Jalladia et al. (2022) have documented the alpha-amylase and alpha-glucosidase activities of *H. cymosum*. To the best of our knowledge, our study is the first study reporting the antidiabetic, anti-inflammatory, and cytotoxicity potentials of ZnO-NPs synthesized from the aqueous extract of *H. cymosum*. The investigation entails using aqueous extracts of South African *H. cymosum* species to synthesize ZnO-NPs from zinc nitrate hydrate. After that, detailed characterization was carried out on the synthesized ZnO-NPs, and their biological activities were investigated.

## Materials and methods

### Plant collection and extract preparation

*Helichrysum cymosum* shoots were harvested in the garden of the Cape Peninsula University of Technology, Bellville Campus, Cape Town, South Africa. The plant was authenticated by a botanist (P. Dryfhout) with voucher number 3708 and stored in the herbarium at the Department of Horticultural Sciences, Cape Peninsula University of Technology, Western Cape, South Africa. Afterwards, the plant was thoroughly washed with distilled water and dried in an oven at 40 °C, then crushing using an electric grinder. The aqueous extract was obtained by boiling 100 g/L at 100 °C for 30 min and then allowed to cool at room temperature. The plant extract was then filtered using a Whatman No. 1 filter paper (Sadiq et al. 2021). The filtrate extract of *H. cymosum* was then stored at 4 °C for subsequent use.

### Biosynthesis of ZnO-NPs of aqueous extract of *H*. *cymosum*

**Fig. 1:**
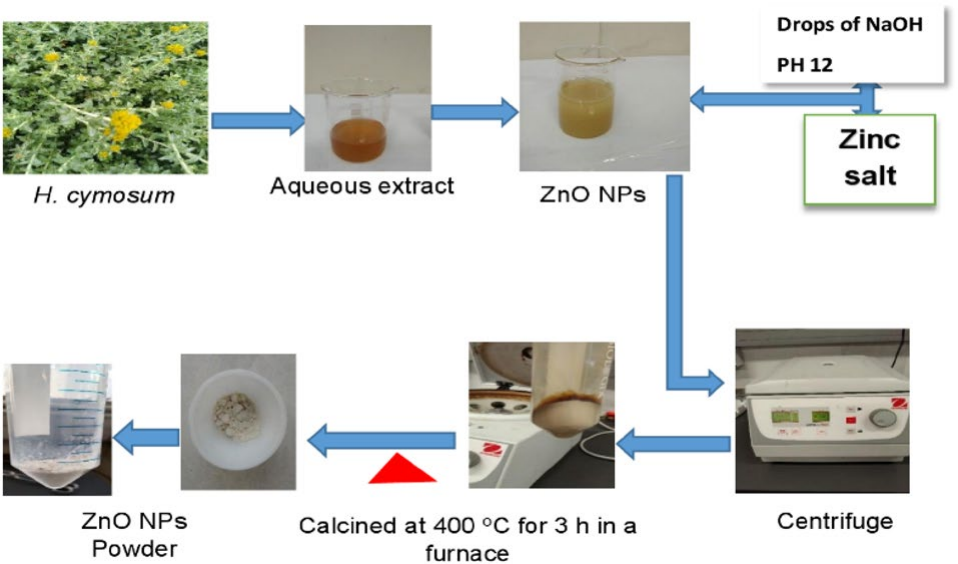
Pictorial illustration of the synthesis process of ZnO-NPs from aqueous extract *H. cymosum* shoots

The biosynthesis of ZnO-NPs was carried out as described by Ifeanyichuku et al. (2020), as illustrated in Fig. 1. Briefly, 9.47 g of zinc nitrate hydrate salts 0.1 M was dissolved in 500 mL of double distilled water and stirred till the complete dissolution of the salt. A volume of 500 ml of *H*. *cymosum* extract was added to the Zn salt mixture and allowed to stir until all the salt was dissolved, followed by 2 M NaOH to adjust the pH to 12 (Issam et al. 2021). The entire mixture was stirred for 3 h at 60 °C. A pale-yellow precipitate was formed, and the mixture was then allowed overnight at room temperature for complete synthesis. The precipitate formed was then collected, centrifuged at 6000 rpm, and washed 3 times using distilled water to remove impurities. The ZnO-NPs were left overnight to dry in an oven at 80 °C, and the resulting powder was further calcined at 400 °C for 3 h in a furnace. A white powder was obtained and crushed using a mortar and pestle to fine powder, and later used for characterization. The ZnO-NPs samples for cell culture analysis were prepared by reconstituting in dimethyl sulfoxide (DMSO) at a concentration of 100 mg/mL, and the mixture was then sonicated for proper dissolution and stored at 4 °C until used.

### Characterization of synthesized ZnO-NPs

Ultraviolet–visible (UV–Vis) spectroscopy (BMG LABTECH-SPECTROstar-Nano, Germany) at spectra range from 280 to 500 nm was used to confirm the formation of ZnO-NPs after synthesis. Origin Pro 8 software was used to plot the data obtained. Information on the stability and dispersion of the ZnO-NPs were determined Zeta potential {(Nano-zs90, country) equipped with both the Zetasizer and zeta potential}. The morphology and crystalline nature of the ZnO-NPs were evaluated using an X-ray diffractometer (XRD), which provides information about symmetry, size, and shape. The Perkin Elmer Spectrum (Version 10.4.2) ATR-FTIR spectrometer was used to determine the phytochemical compounds involved in the reduction and stabilization of the nanoparticles. FTIR of the ZnO-NPs was performed at a wavelength range of 4000–500 cm^−1^. Scanning electron microscopy (SEM) (JEOL JSM-6360LV, Tokyo, Japan) equipped with energy-dispersive X-ray analysis (EDX) (Noran SIX 200 Energy Dispersive X-ray (JOEL, Ltd, Tokyo, Japan) was used to examine the morphology and microstructure (SEM) and purity and elemental compositions (EDX) of the ZnO-NPs. The shape and size distribution of the synthesized ZnO-NPs were analyzed using Transmission electron microscopy (TEM) (Tecnai T20 TEM, LaB_6_ filament) at 200.0 keV BF mode.

### Cell culture

Human hepatoma-derived C3A hepatocytes were purchased from the American Type Culture Collection (ATCC, Manassas, Virginia, USA) and maintained in 10 cm culture dishes in complete medium [minimal essential medium (MEM) with 1% non-essential amino acids, 10% foetal bovine serum (FBS), and 1% penicillin/streptomycin]. The cells were incubated at 37 °C, with 5% CO_2_ in a humidified environment, and subculture post 90% confluence.

### Cellular antioxidant assay (CAA)

The cellular antioxidant activity of ZnO-NPs was examined on C3A hepatocyte cells. One hundred microliters (100 μL) of aliquots of cells were seeded at a density of 2 × 10^4^ cells/well and left to attach overnight. The ZnO-NPs treatment (15 μg/mL. 63 μg/mL, 31.25 μg/mL and 62.5 μg/mL) was reconstituted in a complete medium, and 100 μM catechin was used as a positive control and incubated for 24 h. Oxidative stress was induced by adding tert-butyl hydroperoxide (TBHP) to the culture/treatment medium at a final concentration of 30 μM and incubated for 2 h. The culture/treatment medium was gently aspirated, and 100 μL staining solution [CellROX^®^ Orange (5 μM) and Hoechst 33342 (5 μg/mL) in PBS with Ca^2+^ and Mg^2+^] was added to each well. Plates were incubated for 30 min (protected from light), and fluorescent micrographs were captured immediately using an ImageXpress Micro XLS Widefield Microscope (Molecular Devices) with a 10 × Plan Fluor objective using DAPI and TRITC (tetramethylrhodamine isothiocyanate) filter cubes. Acquired images were analyzed using the MetaXpress software and Multi-Wavelength Cell Scoring Application Module. Antioxidant activity was determined using the average cellular CellROX^®^ Orange fluorescent intensity.

### Cytotoxicity analysis of ZnO-NPs of *H. cymosum*

The cytotoxic effect of the synthesized ZnO-NPs of *H. cymosum* was assessed on C3A cells. In 96-well plates, 100 μL (5000 cells/well) were seeded and left for 24 h to attach. Five hundred (500) μg/mL of ZnO-NPs dilution was prepared in a complete medium and a 6-point dilution of different concentration was made (15.6 µg/mL, 31.25 µg/mL, 62.5 µg/mL, 125 µg/mL, 250 µg/mL, and 500 µg/mL). Afterwards, 100 μL aliquots from each dilution was added to 100 μL of attached cells in the 96-well plate, thus yielding final concentrations of 7.8 µg/mL, 15.6 µg/mL, 31.25 µg/mL, 62.5 µg/mL, 125 µg/mL, and 250 µg/mL. The treated cellscecells were allowed for 48 h at 37 °C, 5% CO_2_, and 30 μM Melphalan (100 mM stock) was used as a positive control. The treatments were aspirated from the wells, and 100 μL MTT (0.5 mg/mL) in the complete medium was added to each well and cells were incubated for 3 h. Thereafter, 100 μL DMSO was added to each well, and absorbance was measured at 540 nm using a BioTek^®^ PowerWave XS spectrophotometer (Winooski, VT, USA).

### Anti-inflammatory activity using RAW 264.7 mouse macrophages

The anti-inflammatory activity of ZnO-NPs was evaluated following the procedure of Shauli et al. (2023) with slight modification. The RAW 264.7 cells were seeded at a density of 1 × 10^5^ cells per well in a 96-well plate and left overnight to attach. The culture medium was removed, and 50 µL of sample aliquots (diluted in RPMI complete medium) were added to give final concentrations of 50, 100, and 200 μg/mL. Thereafter, 50 μL of LPS (final concentration of 500 μg/mL)-containing medium was added to the corresponding wells, and aminoguanidine (AG) was used as the positive control at 100 μM, followed by incubation for 24 h. The spent culture medium (50 μL) was transferred to a new 96-well plate to quantify nitric oxide (NO) production. Sulfanilamide solution and NED solution were prepared as per the manufacturer's instructions. An aliquot volume of 50 μL sulfanilamide solution was added to the spent culture medium and incubated for 10 min in the dark, at room temperature. Fifty (50) μL NED [N-(1-Naphtyl)-ethylenediamine dihydrochloride] solution was added to each well and further incubated for 5–10 min in the dark at room temperature. Absorbance was measured at 540 nm (BioTek^®^ PowerWave XS spectrophotometer), and a nitrite standard curve (using sodium nitrite dissolved in culture medium) was used to determine the concentration of NO in each sample.

### Alpha-amylase inhibitory activity

Alpha-amylase inhibitory activity was conducted as described by Aladejana et al. (2020), using the 3, 5-dinitrosalicylic acid (DNS) method with slight modifications. Forty microliters (40 μL) of different concentration ranges of ZnO-NPs test samples were placed in test tubes. In the same test tubes, 400 μL of starch solution (0.5 g starch in 50 mL phosphate buffer) and 200 μL α-amylase (4 units/mL) were added and incubated at 35 °C for 10 min to start the reaction. After incubation, 200 μL of the test samples were transferred into new test tubes, and 100 μl of DNS (20 mL of 30 g of sodium potassium tartrate tetrahydrate mixed with 50 mL of 1 g 3, 5-dinitro salicylic acid solution and 20 mL of 2 M NaOH at 90–95 °C) was added to stop the reaction. The solution was then boiled for 15 min and allowed to cool at room temperature. Subsequently, distilled water (900 μL) was added to dilute the solution. Two hundred microliters (200 μL) of sample, blanks, and positive control were prepared and placed in a 96-well plate, and the absorbance was measured at 540 nm using a UV spectrophotometer. The percentage inhibition of the enzyme α-amylase was determined using the following equation:

$$\% \text{alpha-amylase inhibition}  = \frac{(\text{absorbance of control}-\text{ absorbance of test sample})}{\text{absorbance of control}}\times 100$$

### Alpha-glucosidase activity

The alpha-glucosidase activity of ZnO-NPs was assessed according to the procedure described by Erukainure (2018). Briefly, 50 μL of both plant extracts at different concentrations (1000, 500, 250, 100, 50, and 10 μg/mL) and α-glucosidase (1.0 Unit/mL) prepared in phosphate buffer 100 mM at pH 6.8 were placed in a 96-well plate and incubated for 15 min at 37 °C. Afterwards, 100 µL of 5 mM p-nitrophenyl-α-d-glucopyranoside (pNPG) solution prepared in phosphate buffer (100 mM, pH 6.8) was added to the reaction mixture and incubated for an additional 20 min at 37 °C. Acarbose was used as a positive control, and the absorbance was measured at 405 nm. The percentage inhibition was calculated with the formula below.

$$\% \text{alpha-glucosidase inhibition} = \frac{(\text{absorbance of control}-\text{ absorbance of test sample})}{\text{absorbance of control}}\times 100$$

### Glucose uptake and utilization

With modifications, glucose uptake and utilization were evaluated using C3A hepatocytes and L6 myocytes following the procedure in van de Venter et al. (2008). Both cells (C3A and L6) were seeded in 96-well plates (2 × 10^4^ cells/well, 100 μL aliquots) and left overnight to attach. Various concentrations of treatments were prepared in a complete medium and added to the cells, followed by incubation for 24 h and 48 h for each cell type. After incubation, cell culture/treatment medium (5 μL for C3A; 10 μL for L6) was removed from the respective plates and transferred into new 96-well plates (A), which were sealed and stored at − 20 °C until required. The rest of the medium was aspirated, and cells washed with 100 μL PBS and 25 μL incubation buffer (RPMI-1640 diluted with PBS containing 0.1% bovine serum albumin (BSA) to a final glucose concentration of 8 mM) were added to cells (C3A and L6). Insulin (1 μg/mL) was used as a positive control, and the cells were then incubated for another 4 h. Some culture medium (5 μL) was transferred to a new 96-well (plate B). Afterwards, 200 μL of glucose oxidase reagent [3 mM phenol, 0.4 mM 4-amino antipyrine, 0.25 mM EDTA, and 2.5 U/mL horseradish peroxidase in 0.5 M PBS (pH 7.0) with 1 mU/mL glucose oxidase from *Aspergillus niger*] was added to the plates (A and B), respectively, and incubated for 15 min at room temperature. Cell-free wells containing incubation buffer and complete culture medium were used as glucose standards. The absorbance was then measured at 510 nm using a BioTek^®^ PowerWave XS spectrophotometer (Winooski, VT, USA. Glucose uptake and consumption were determined as a function of the concentration of glucose (mM) remaining and expressed as the difference between the mean of the standard and test samples. The MTT assay was further used to determine cell viability.

### Pancreatic lipase inhibition

Pancreatic lipase inhibitory activity of ZnO-NPs was performed following the procedure described by Pringle et al. (2021). A volume of 10 μL of sample at different concentrations and 5 μL of porcine pancreatic lipase enzyme (100 mg mL^−1^ prepared in 100 mM Tris–HCl, pH 8.0) was pre-incubated at 37 °C for 15 min. Afterwards, 170 μL of the substrate (*p*-nitrophenyl palmitate (pNPP) [1 mg/mL in isopropanol) with reaction buffer (gum arabic (1 mgmL^−1^), sodium deoxycholate (2 mg mL^−1^), and Triton X-100 (5 μL per mL) prepared in 100 mM Tris–HCl (pH 8.0)] was added to the mixture containing extracts and enzyme and incubated at 37 °C for 25 min. Pancreatic lipase activity was then determined by measuring the absorbance at 405 nm using a BioTek^®^ PowerWave XS spectrophotometer, and 100 μM Orlistat was prepared and used as a positive control. The percentage of pancreatic lipase inhibition was calculated according to the following equation:

$$\% \text{Lipase inhibition} = \frac{\text{A}405\text{nm of blank}-\text{ A}405\text{nm of test sample})}{\text{A}405\text{nm of blank}}\times 100$$

### Statistical analysis

Results are expressed as a mean ± standard error of the mean. Differences between the means were determined by one-way analysis of variance (ANOVA) followed by Bonferroni post-test. All analyses were performed with GraphPad Prism 5. The difference between the mean values of *p* < 0.05 was considered statistically significant.

## Results

**Fig. 2:**
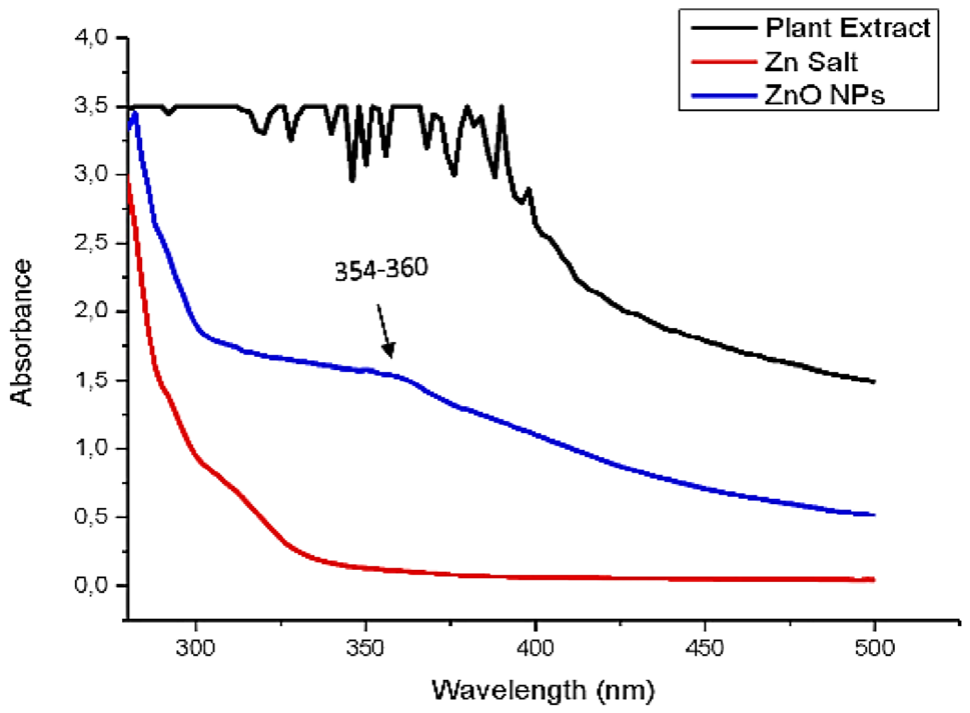
UV–Vis spectroscopy analysis of ZnO-NPs synthesized form aqueous extract of *H*. *cymosum*

The UV–Vis spectrometry analysis was the preliminary step to confirm the synthesis of zinc metal oxide nanoparticles by the surface plasmon resonance band (SPR) (Issam et al. 2021). The UV–Vis spectrum was scaled on a wavelength range from 280 to 500 nm for *H. cymosum*-mediated ZnO-NPs, as shown in Fig. 2. The results show a broad peak range of 354–360 nm, confirming a successful formation of ZnO-NPs with a maximum absorption peak observed at 360 nm.

### Scanning electron microscopy (SEM) and energy-dispersive X-ray (EDX) of *H. cymosum* ZnO-NPs

**Fig. 3:**
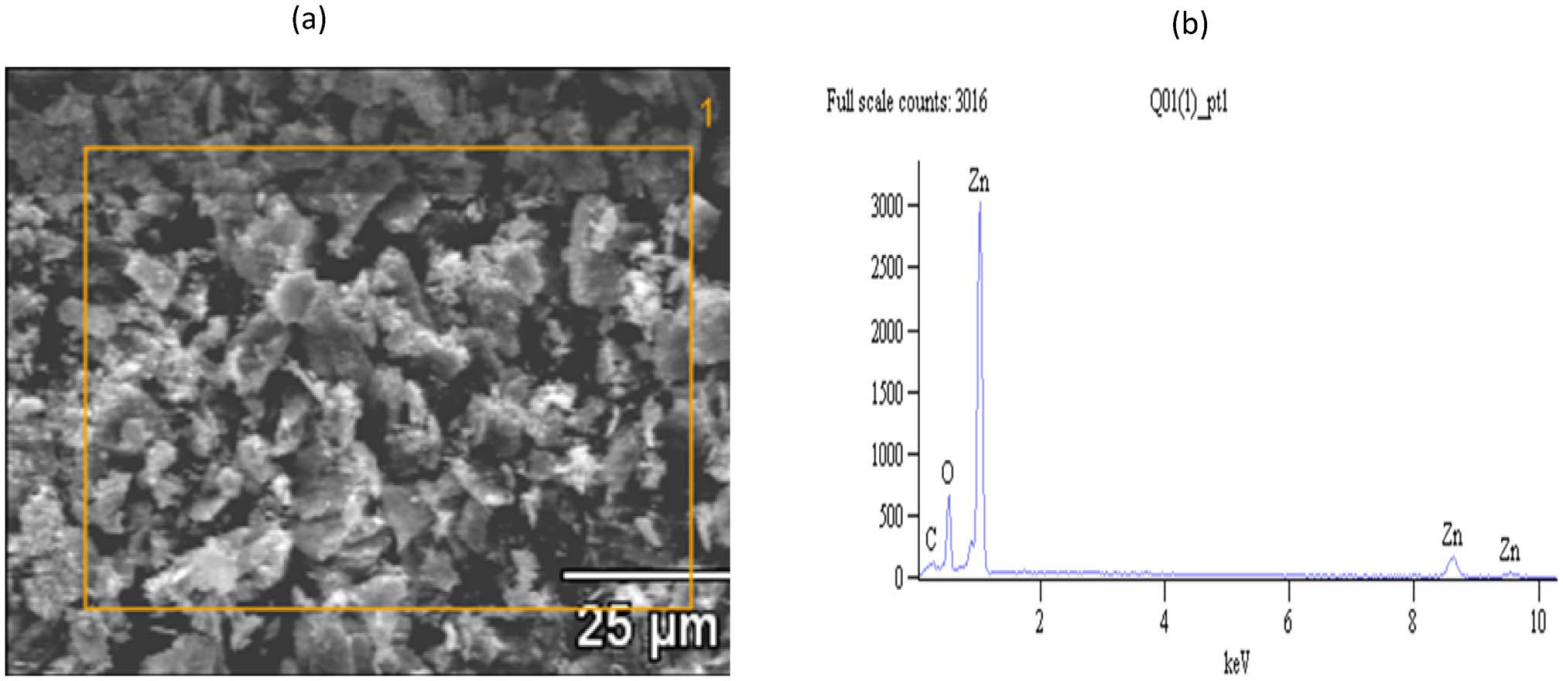
Scanning electron microscopy (**a**) and EDX spectrum (**b**) analysis of aqueous extract of *H. cymosum* synthesized ZnO-NPs

The SEM analysis was used to study the surface morphology of synthesized ZnO-NPs from *H. cymosum* aqueous extract, and the results are represented in Fig. 3a. The images showed an aggregate of closely packed irregular hexagonal shapes of the ZnO-NPs. The EDX results depicted in Fig. 3b reveal two fundamental elements, Zn and O, on the spectra of *H. cymosum*-mediated ZnO-NPs. An additional peak of C was identified and could be associated with the bioactive compound capping during ZnO-NPs formation.

### TEM analysis

**Fig. 4:**
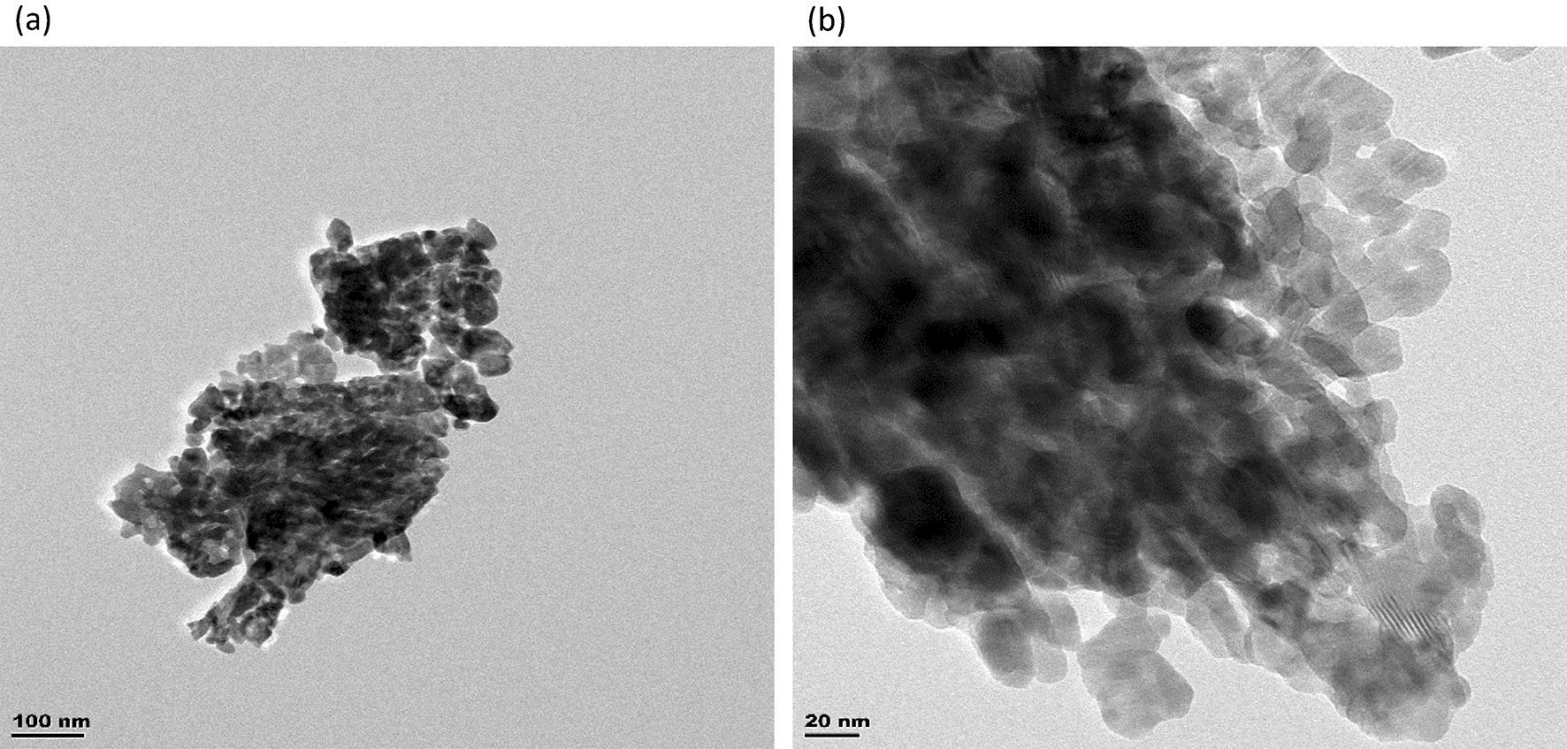
TEM analysis (**a** and **b**) of aqueous extracts of *H. cymosum*-mediated zinc oxide nanoparticles

The surface morphology of the ZnO-NPs was interpreted using TEM at different magnifications, as shown by the micrographs in Fig. 4*. The resulting images showed an agglomeration of nanoparticles with dark spots. The nanoparticles were spherical and hexagonal, with an average dimension of 28.05–58.3 nm. The dark areas observed might have resulted from the agglomeration of the NP during synthesis, which corroborates with observations from other studies in the literature (Mbenga et al. 2022; Mkhize et al. 2022).

### Zeta potential

**Fig. 5:**
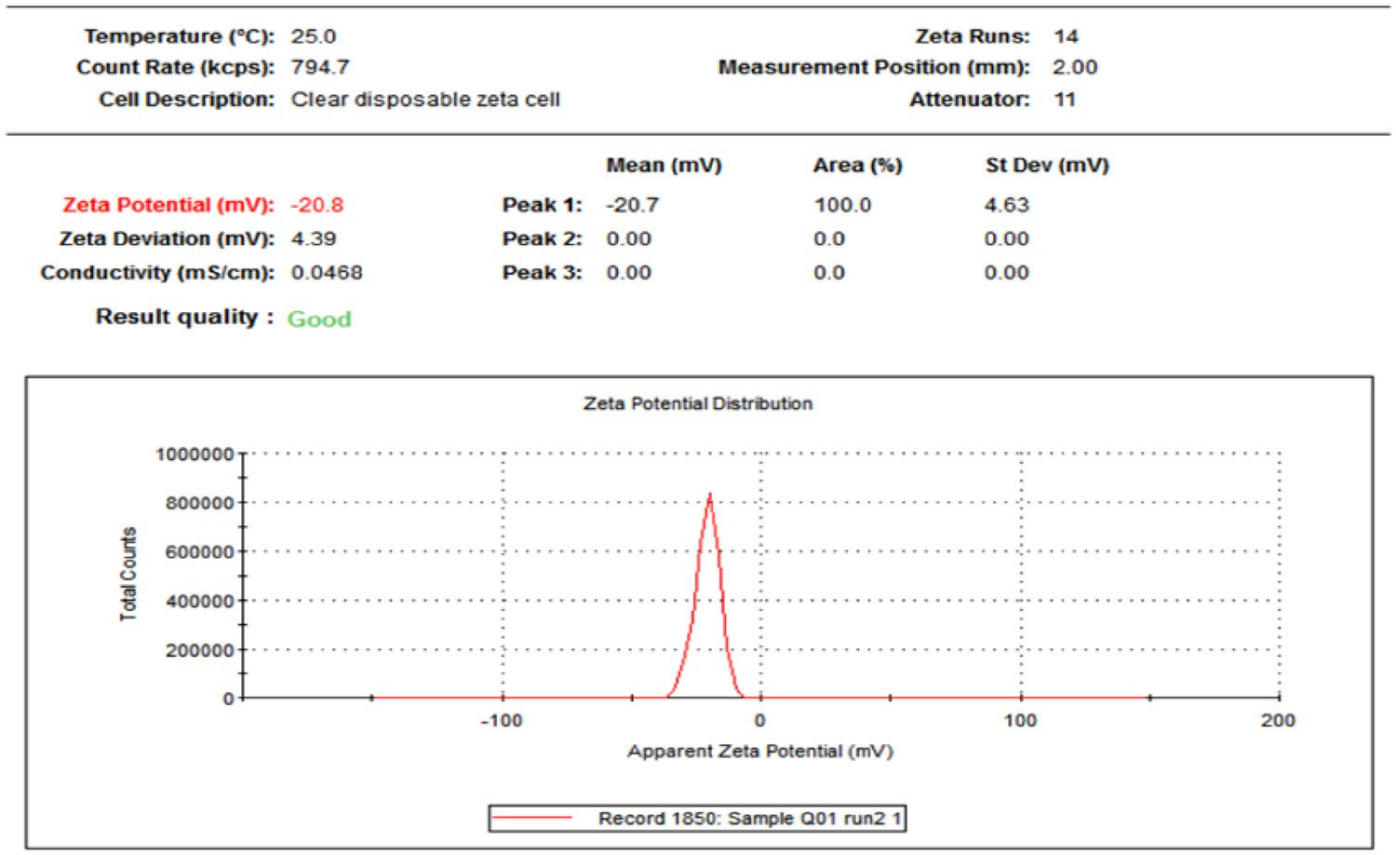
Zeta potential (mV) of ZnO-NPs synthesized from aqueous extract of *H. cymosum* shoot

The dispersion stability of nanofluid was measured by the absolute value of zeta potential (Fig. 5). In the present study, the synthesized ZnO-NPs of *H. cymosum* showed a negative charge with good stability of − 20.8 mV.

### FTIR analysis of ZnO-NPs

**Fig. 6:**
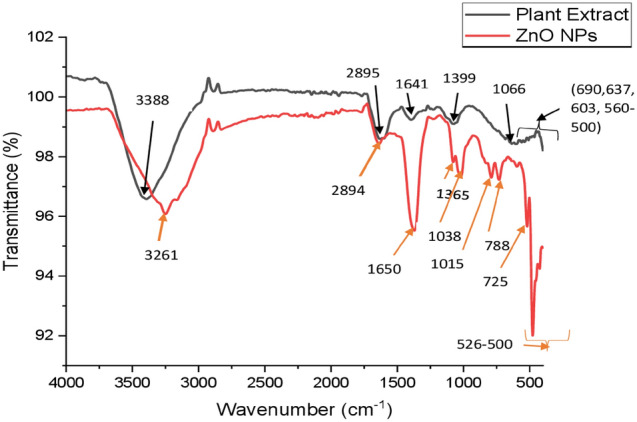
FTIR spectrum of aqueous extract of *H. cymosum*-mediated ZnO-NPs. The different peaks represent the identified functional groups

The surface functional groups of the synthesized ZnO-NPs of *H. cymosum* were investigated using FTIR analysis at wavelengths of 4000–500 cm^−1^. Different peaks characterizing biomolecules were identified from the spectra (Fig. 6). The results reveal a broad peak at 3388 and 3261 cm^−1^corresponding to the O–H-stretching vibration and to the alcohol and phenols functional group, or water molecules present in the extract (Bayrami et al. 2018; Mkhize et al. 2022; Unni et al. 2022). The absorption peak of 2895 and 2894 cm^−1^ signifies the stretching for C–H bonds (alkanes), peak 1650 cm^−1^, 1641 cm^−1^ C=O, primary amide, 1399 cm^−1^ (NO_2_). From 1083 cm^−1^,1066 cm^−1^, and 1015 cm^−1^ are allocated to the presence of –N–H, –C–O, and =C–H and are linked to aliphatic amine, phenol, and carboxylic acid). The peak ranges from 788 cm^−1^, 725 cm^−1^, 690 cm^−1^, 637 cm^−1^, 603 cm^−1^, and 560–500 cm^−1^) are indicative of the N–H characteristic of amines; these spectrum peaks corroborate the formation of ZnO-NPs (Donga and Chanda 2022). The above peaks belong to the vibration stretch of the plant extract, and ZnO-NPs oven-dried at 80° C are indicative of the biomolecule present at the surface of ZnO-NPs responsible for capping and stabilizing the bio-fabricated nanoparticles. The calcined FTIR spectra at 400 °C also retain some peaks which could be allotted to the following functional group (Bayrami et al. 2018, Unni et al. 2022). According to Akintelu et al. (2022), biomolecule functional groups, such as –O–H, C=O, C=C, C–N, C–H, and N–H, are good reducing agents for ZnO-NPs synthesis.

### X-ray diffraction (XRD)

**Fig. 7:**
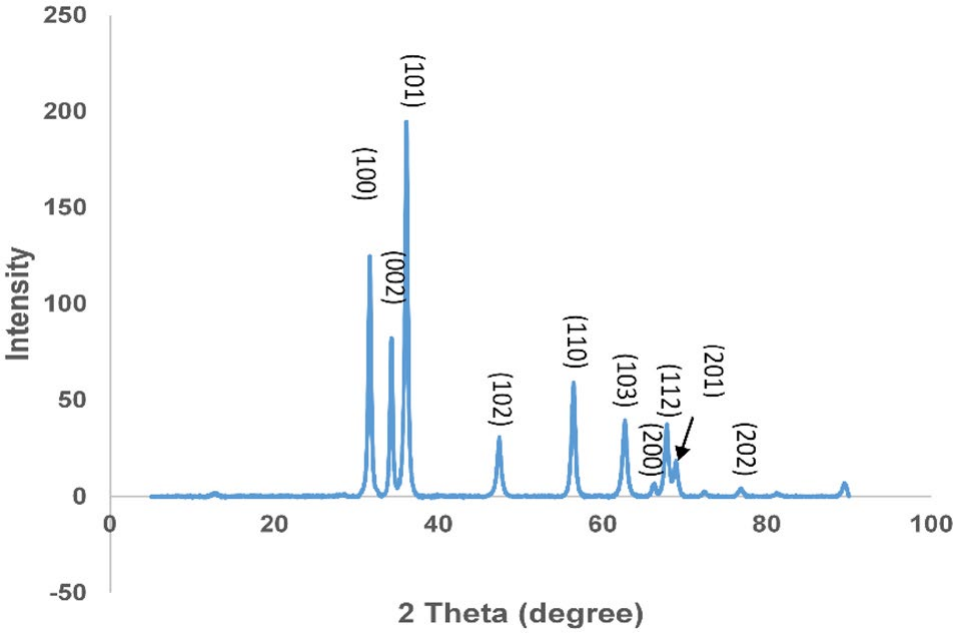
XRD spectra of ZnO-NPs of aqueous extract of *H. cymosum*

Figure 7 depicts the XRD patterns of ZnO-NPs of *H. cymosum*. The results reveal prominent diffraction peaks at 2θ = 31.64°, 34.31°, 36.11°, 47.46°, 56.48°, 62.71°, 66.17°, 67.84°, 68.99°, 77.17°, 89.57°, which correspond to the crystal reflection planes 100, 002, 101,102, 110,103, 200, 112, and 202 of the hexagonal structure of ZnO phase. The matching of the peaks was according to the reported standard values in the joint committee on powder diffraction standards (JCPDS) card No: 036-1451 (Obeizi et al. 2020; Darezereshki et al. 2021).

### Cytotoxicity assay (MTT)

**Fig. 8:**
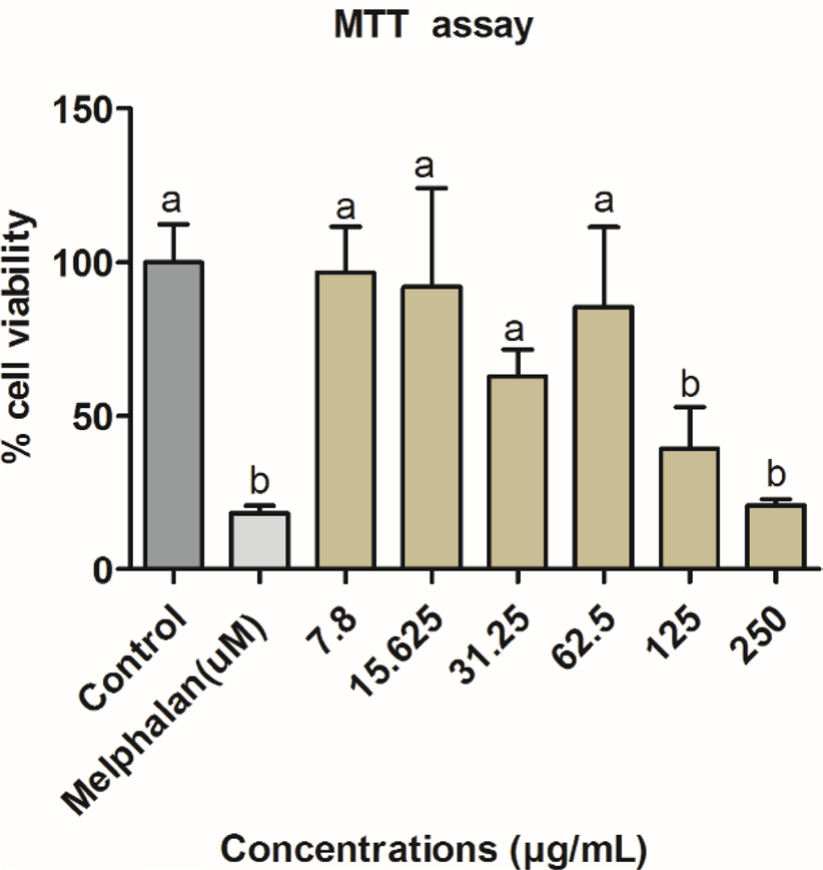
The % cell viability of C3A hepatocytes cells treated with *H. cymosum* ZnO-NPs for 48 h. Each bar represent mean ± SD of four replicate wells, and melphalan was used as a positive control. Bars with different letters present significant different (*p* < 0.05) compared to the untreated control

*H. cymosum* synthesized ZnO-NPs were evaluated for toxicity on C3A cells; the results are represented in Fig. 8. The cells were treated for 48 h with different concentrations (7.8 µg/mL, 15.6 µg/mL, 31.25 µg/mL, 62.5 µg/mL, 125 µg/mL, and 250 µg/mL). The synthesized ZnO-NPs at lower concentrations (7.8—62.5 µg/mL) did not cause any substantial change in cell viability compared to the control, but a significant change (*p* < 0.05) was noted compared to the positive control. Meanwhile, at higher concentrations (125 µg/mL and 250 µg/mL), there was a sharp decline in cell viability which was significant (*p* < 0.05) compared to the control. In summary, the ZnO-NPs were safe up to 62.5 µg/mL, and beyond this limit, a cytotoxic effect was observed.

### Cellular antioxidant activities (CAA)

**Fig. 9:**
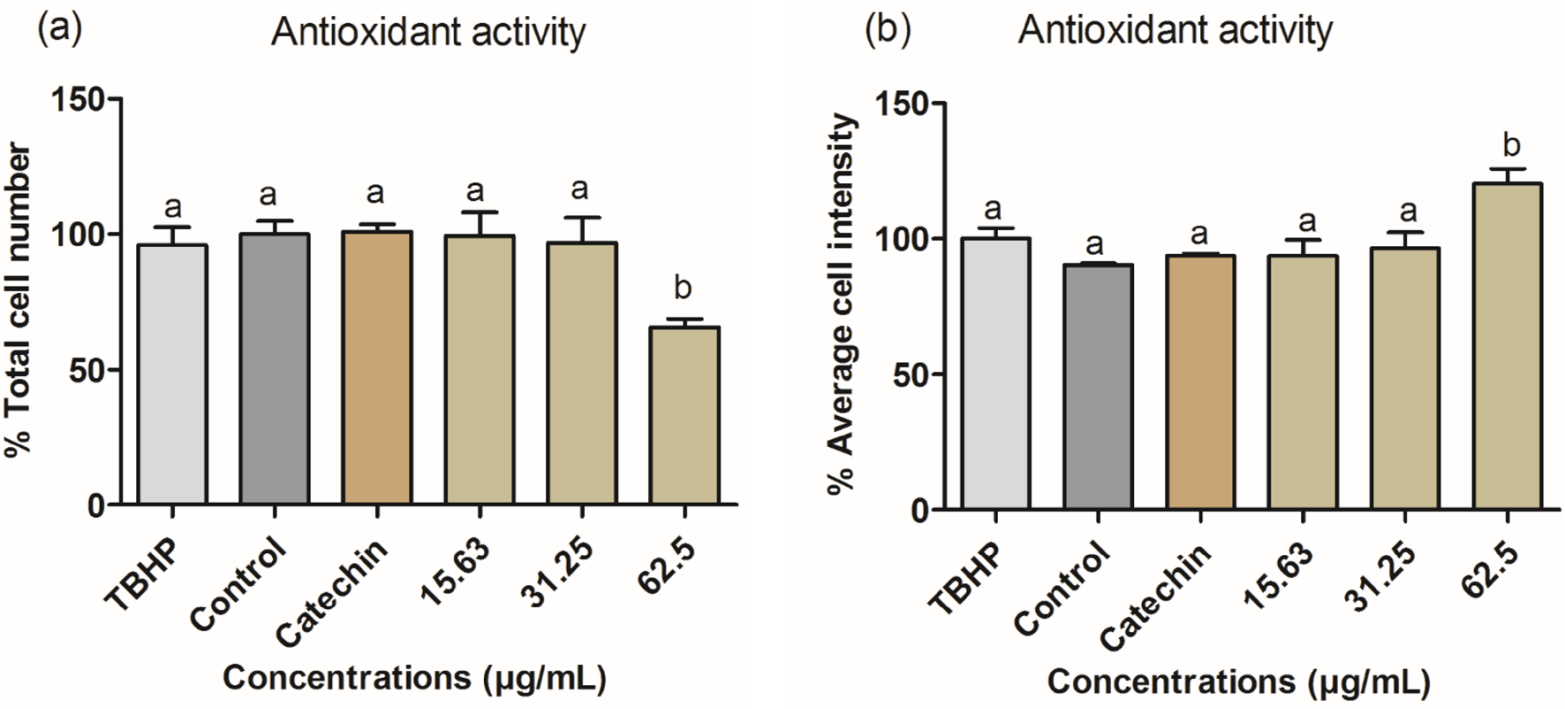
% total number of cells (**a**) and % average cell intensity (**b**) after 24 h of treatment of C3A cells with ZnO-NPs. Error bars represent the mean ± SD of four replicate wells, Catechin (100 µM) was used as a positive control and significant toxicity (*p* < 0.05 compared to TBHP). Different letters above the bar for each given concentration compared to the control are significantly different (*p* < 0.05)

After treatment, the cellular antioxidant activity of the synthesized ZnO-NPs showed significant toxicity at 62.5 μg/mL (Fig. 9a). Therefore, the antioxidant capacity at this concentration cannot be reliably interpreted. In addition, the ROS level also increased at a similar concentration (62.5 μg/mL) as indicated by the average cell intensities. The remaining concentrations (15.63 and 31.25 μg/mL) did not induce CAA activity (Fig. 9b).

### Anti-inflammatory activities

**Fig. 10:**
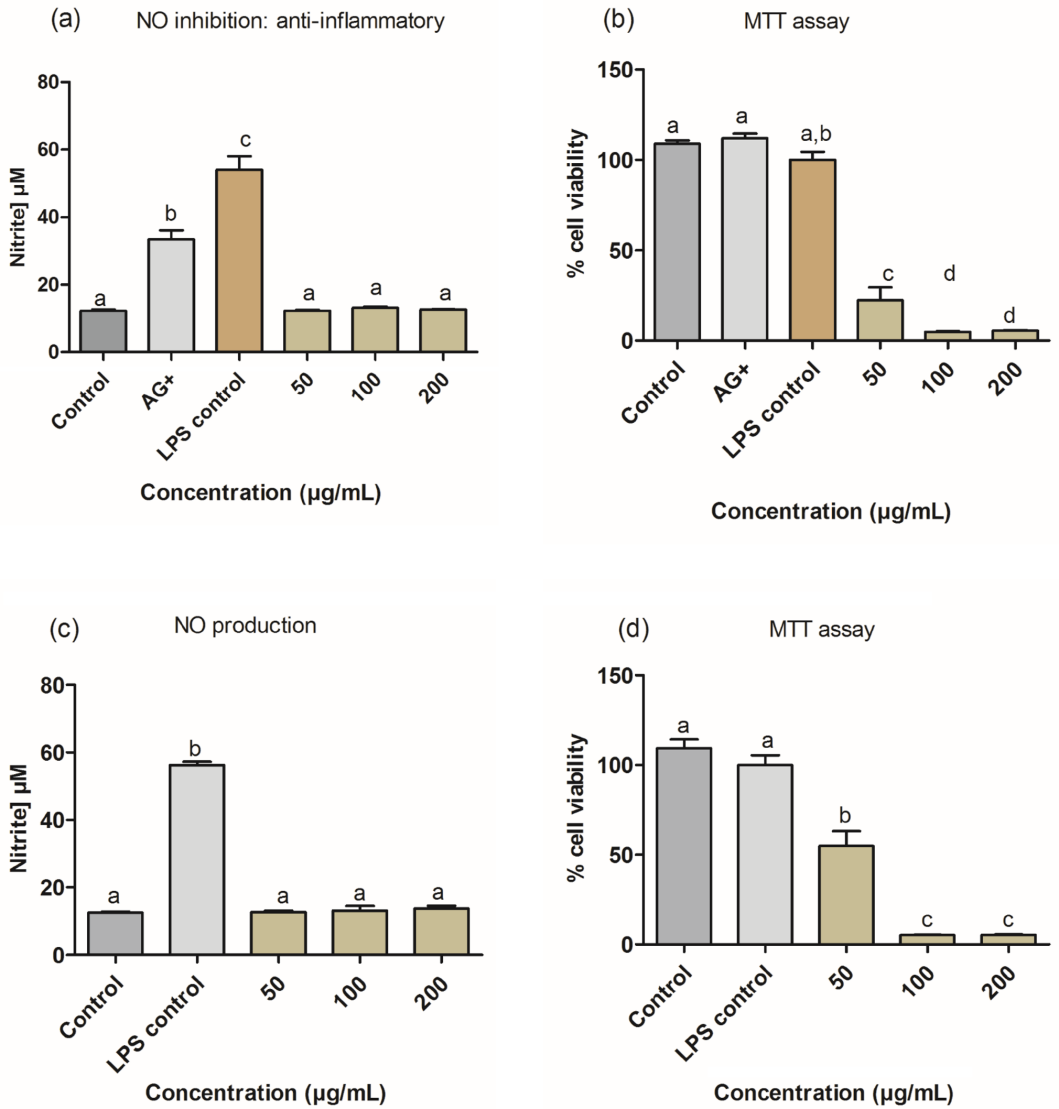
Nitric oxide production (**a**) and inhibition of LPS-activated macrophages (**c**), and their corresponding cell viability (%) (**b** and **d** respectively) after 24 h treatments with ZnO-NPs. Error bars indicate the mean ± SD of four replicate wells. Bars with different letters present significant different (*p* < 0.05) compared to the AG-control and untreated control

In Fig. 10, the anti-inflammatory activity is indicated by the decrease in nitrite concentration in response to LPS activation of RAW macrophages with no effect on cell viability, as seen with the AG-treated cells which significantly (*p* < 0.05) decreased the nitrites less than 40% (Fig. 10a) with increase cell viability above 100% (Fig. 10b). This shows a better anti-inflammatory activity compared to the ZnO-NPs treatments. The findings showed that the 50, 100, and 200 μg/mL concentrations of ZnO-NPs could substantially decrease NO production (Fig. 10a and c) but are cytotoxic at all tested concentrations (Fig. 10b and d) with a significant reduction (*p* < 0.05) in cell viability. Therefore, the NO results were not considered.

### Glucose uptake and glucose utilization

**Fig. 11:**
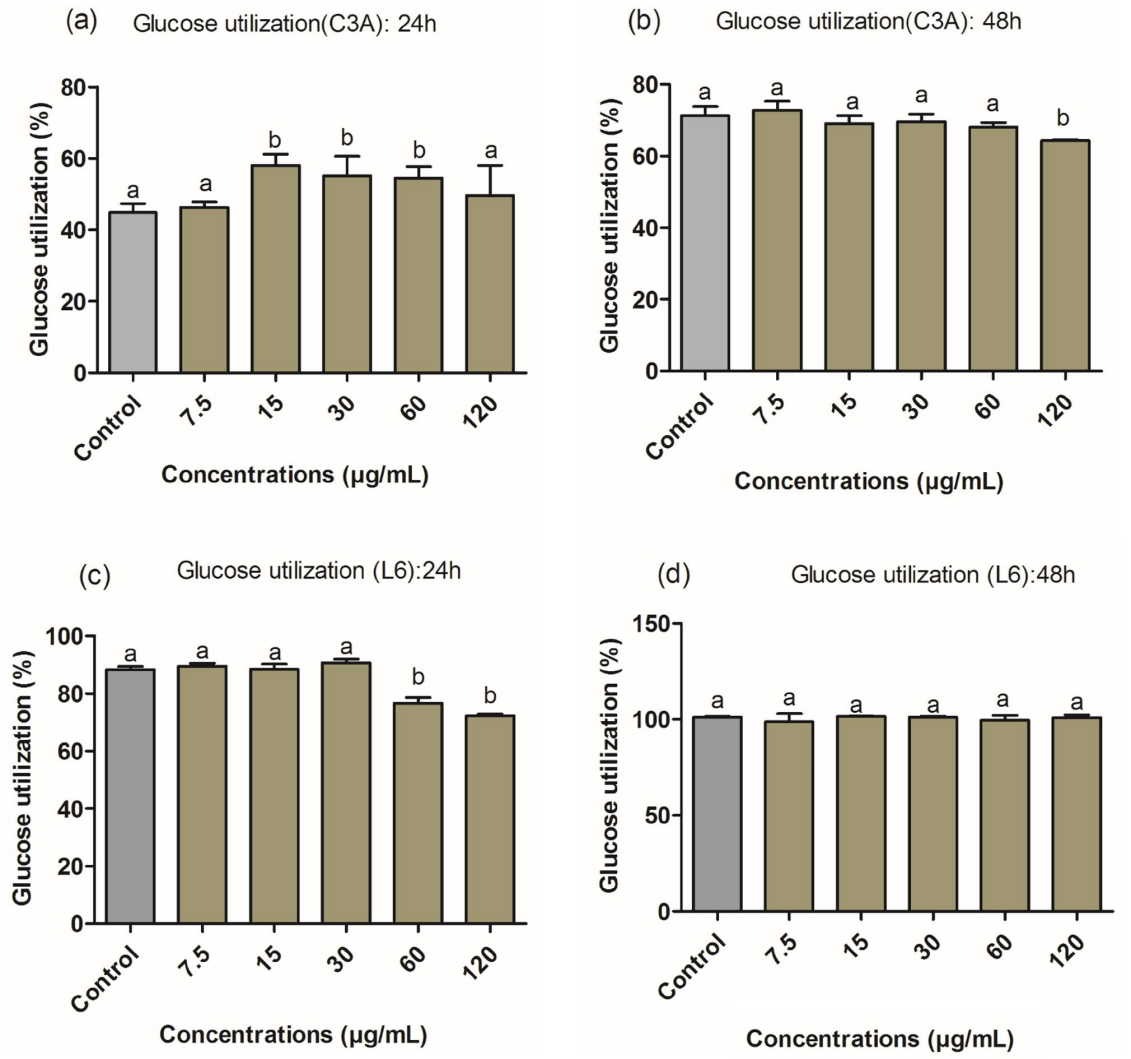
Glucose utilization (%) after 24 h (**a** and **c**) and 48 h (**b** and **d**) of treatment by *H. cymosum* ZnO-NPs in C3A hepatocytes and L6 myocytes. Results were normalized to cell viability as determined using the MTT assay. Error bars indicate the standard deviation of the mean of four replicates wells. Letter above the bars indicates a significant difference (*p* < 0.05) compared to the control

**Fig. 12:**
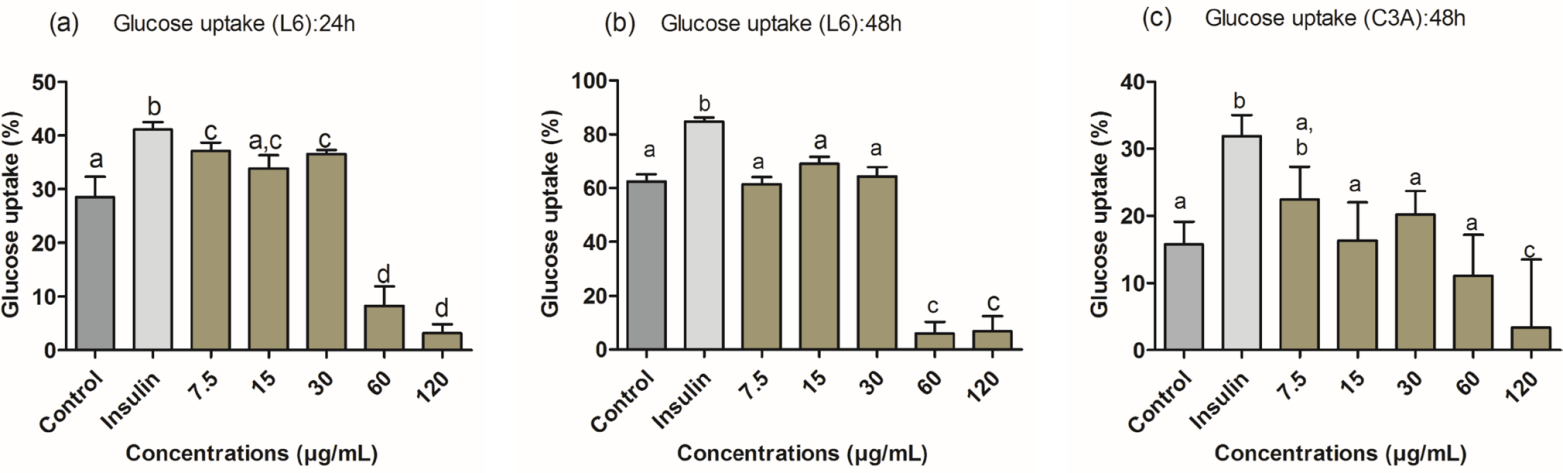
Glucose uptake (%) by L6 myocytes and in C3A hepatocytes, following 24 h (**a**) and 48 h (**b** and **c**) pre-treatment with *H. cymosum* ZnO-NPs. Results were normalized to cell viability as determined using the MTT assay. Error bars indicate the standard deviation of the mean of four replicates wells. Different letters on the bars signify statistics significance (*p* < 0.05) compared to the untreated control

The glucose uptake and utilization by C3A hepatocyte and L6 myocyte cells were used to estimate the antidiabetic activity of ZnO-NPs of *H. cymosum*. The C3A cells exposed to ZnO-NPs showed a significant increase in glucose utilization at 15, 30, and 60 µg/mL within 24 h of treatment Fig. 11a. After 48 h of treatment, ZnO-NPs exhibited an equal rate of glucose utilization from 7.5 to 60 µg/mL compared to the control Fig. 11b, and cytotoxicity was observed at 120 µg/mL for both 24 h and 48 h (Fig. 11a and b). The MTT was used to normalize glucose uptake and utilization data to compensate for any differences in cell numbers due to exposure to treatment. The L6-treated cells within 24 h showed a small but significant increase in glucose utilization at 30 µg/mL Fig. 11c. After 48 h, the cells utilized all the available glucose (Fig. 11d). As a result, no differences were observed between the treatments. Cytotoxicity was seen at the highest concentrations (60 and 120 µg/mL) for 24 and 48 h (Fig. 11c and d). Figure 12a–c shows that exposure to ZnO-NPs increased glucose uptake at concentrations of 7.5, 15, and 30 µg/mL within 24 h and 15 and 30 µg/mL after 48 h. Cytotoxicity was observed at (60 and 120 µg/mL) in (Fig. 12a and b) and 120 µg/mL in Fig. 12c.

### Pancreatic lipase inhibition

**Fig. 13:**
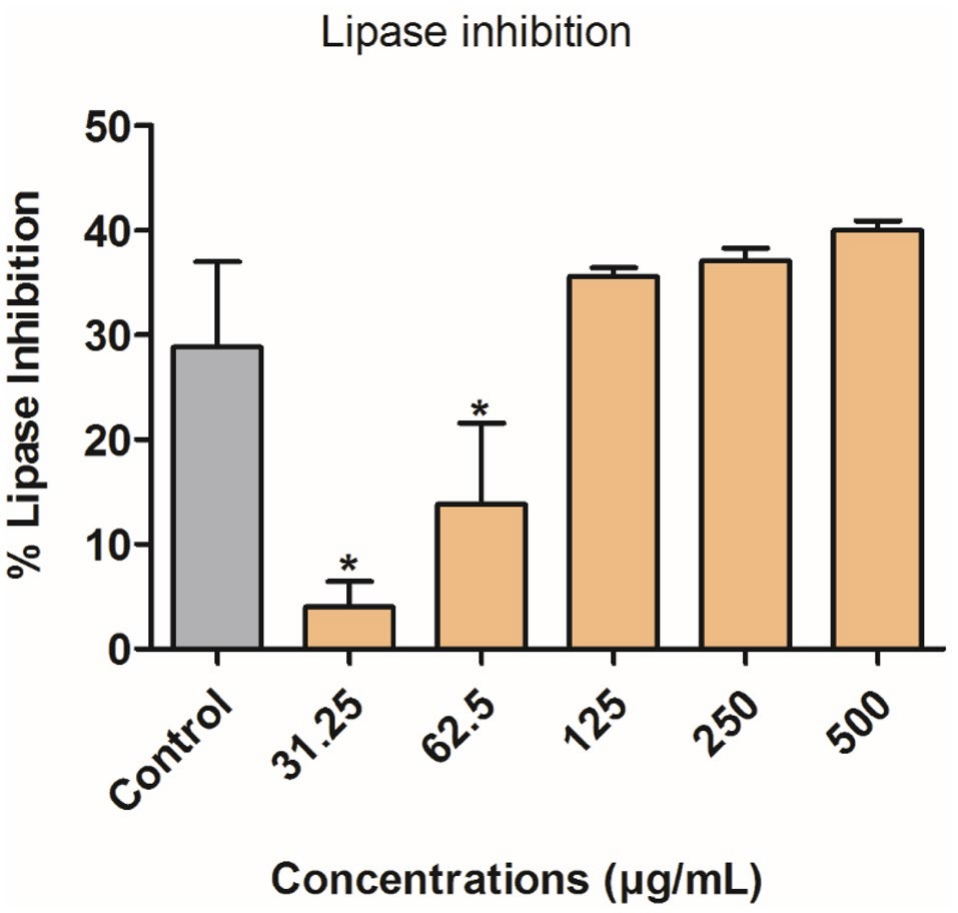
Pancreatic lipase inhibition of *H. cymosum* ZnO-NPs. Error bars indicate the standard deviation of the mean of four replicates. The * on the bars indicate significant difference (*p* < 0.05) compared to the control orlistat (100 µM)

Pancreatic lipase inhibition is a promising mechanism to combat obesity and hypertriglyceridemia (Lunagariya et al. 2014). Inhibition was minimal at 31.25 and 62.5 µg/mL but gradually increased from 125 to 500 µg/mL compared to the control (Fig. 13). However, statistical significance was not achieved at 125–500 µg/mL compared to the control (Orlistat: 100 µM).

### Alpha-glucosidase and alpha-amylase

**Fig. 14:**
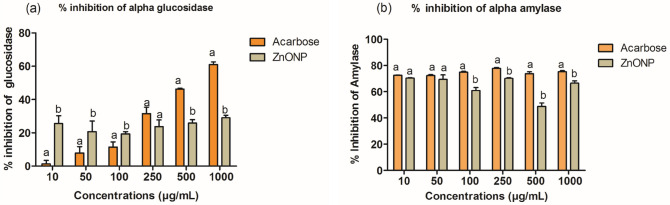
α-glucosidase (**a**) and α-amylase (**b**) inhibitory activities of *H. cymosum* ZnO-NPs. Data were express as mean ± SD; *n* = 4. The bars with different letters shows significantly different (*p* < 0.05). The % inhibition was compared to the control acarbose

The antidiabetic effect of the synthesized ZnO-NPs of *H.cymosum* was evaluated using alpha-glucosidase and alpha-amylase inhibitory assay. A standard drug-acarbose was used as a positive control. The ZnO-NPs inhibited alpha-glucosidase significantly (*p* < 0.05) from 10 to 100 µg/mL. Subsequently, the control (acarbose) showed higher inhibition than the tested ZnO-NPs from 250 to 1000 µg/mL (Fig. 14a). Figure 14b indicates that ZnO-NPs exhibited a comparable level of inhibition of alpha-amylase at 10–50 µg/mL to that of the control with no significant difference. The higher concentrations (100–1000 µg/mL) showed a slight decline compared to the control with a significant difference. The present findings reveal that the lower concentrations of ZnO-NPs of *H. cymosum* have a higher anti-hyperglycemic effect and could be used in mitigating blood glucose levels.

## Discussion

Nanoparticles have received substantial attention due to their unique size range, making them suitable for medical and biological applications (Tang et al. 2019; Siddiqui et al. 2020; Ifeanyichukwu et al. 2020). In the current study, *H. cymosum*-mediated zinc oxide nanoparticles were characterized using UV–Vis spectroscopy, Scanning electron microscope (SEM), Energy-dispersive X-ray spectroscopy (EDX), X-ray diffraction (XRD), Transmission electron microscope (TEM), and zeta potential. The UV–Vis (Fig. 2) showed a successful formation of ZnO-NPs ranging from 354 to 360 nm, attaining its maximum at 360, which aligns with similar peaks reported in the literature (Ifeanyichukwu et al. 2020). The SEM analysis (Fig. 3a) showed a cluster of irregular hexagonal shapes of the ZnO-NPs, suggesting that the high surface area of the NPs might have caused them to stick together (Sundrarajan et al. 2015; Mkhize et al. 2022; Unni et al. 2022). The compounds Zn, O, and C were identified from EDX analysis (Fig. 3b). The presence of the C peak could be associated with the bioactive compound capping ZnO-NPs during formation. Similarly, Bayrami et al. (2020) reported Zn, O, and C on ZnO-NPs of *Urtica dioica* extract. Other studies have reported that Zn and O are elemental composition of ZnO-NPs, signifying the purity of the elemental compositions present in ZnO-NPs (Bayrami et al. 2020; Mbenga et al. 2022).

The TEM analysis also reveals a cluster of spherical and hexagonal-shaped nanoparticles with an average dimension of 28.05–58.3 nm (Fig. 4a and b). The dark areas observed might have resulted from the agglomeration of the NP during synthesis, corroborating other studies (Mbenga et al. 2022; Mkhize et al. 2022). The zeta potential showed an excellent dispersion stability of nanofluids at − 20.8 mV (Fig. 5). The results revealed that the negatively charged molecules found on the ZnO-NPs are involved in capping and stabilizing the nanoparticles (Alarmdari et al. 2020; Mkhize et al. 2022). In a study by Mohana and Sumathi (2020), the zeta potential of *Agaricus bisporus*-mediated ZnO-NPs was − 20.5 mV with good stability and aligns with our present finding. Mkhize et al. (2022) also reported − 23 mV and good stability. Previous studies have established that nanoparticles with − 25 mV and + 25 mV are likely to be more stable (Mahobia et al. 2016; Mkhize et al. 2022). However, the colloidal dispersion stability with absolute zeta potential has been reported to be 30 mV for ZnO-NPs. Thus, the lower colloidal stability of the ZnO-NPs dispersion could have influenced the possible aggregation of the nanoparticles (Hidayat 2018). In addition, the nanoparticles’ composition and medium of dispersion are known to influence the surface charge of the particles (Mahobia et al. 2016).

The FTIR peaks reveal the presence of important functional groups capping the ZnO-NPs, such as alkanes, amines, carboxylic acids, and phenols (Fig. 6) (Bayrami et al. 2018; Mkhize et al. 2022; Unni et al. 2022; Donga and Chanda 2022). These functional groups played a positive role in the reduction of zinc (Zn^2+^) and stabilizing the ZnO-NPs during synthesis (Ochieng et al. 2015; Ifeanyichukwu et al. 2020; Aldeen et al. 2022). The XRD analysis reveals prominent peaks which correspond to the crystal reflection plane of the hexagonal structure of ZnO-NPs phase (Fig. 7). The high-intensity diffraction peaks signify the formation of the superior crystalline quality of the nanoparticles (Obeizi et al. 2020; Alrajhi et al. 2023). The crystalline quality and purity of the ZnO-NPs might be influenced by the presence of a stabilizing agent capping the surface of the ZnO-NPs of *H. cymosum* (Alrajhi et al. 2021). Our finding aligns with other reported XRD peaks in previous studies, confirming the formation of a crystalline monoclinic structure (Bhattacharya et al. 2020; Obeizi et al. 2020; Alrajhi et al. 2023; Sadiq et al. 2021; Rashwan et al. 2022). Zinc oxide nanoparticles, amongst other metal oxides, play an important role in many enzymatic and cellular activities and have been well investigated and applied in diseases, such as cancer, diabetes, etc. (Tang 2019; Ahmed 2022).

Diabetes mellitus (DM) is a metabolic disorder arising from limited insulin secretion or action during carbohydrate, fat, and protein metabolism, resulting in a rise in blood sugar levels (hyperglycemia) (Rehana et al. 2017; Kifle and Enyew 2020; Gadoa et al. 2022). There are several antidiabetic drugs; however, they are associated with side-effects (Haase et al. 2008; Gadoa et al. 2022). One of the important ways to manage DM is by targeting and decelerating the hydrolyzing enzyme (α-glucosidase and α-amylase) activity. Enzymes such as α-glucosidase and α-amylase play a major role in carbohydrate metabolism and absorption. Therefore, the inhibition of the activities of these enzymes could help lessen the burden of post-prandial hyperglycemia (Rehana et al. 2017). Several hyperglycemic drugs, including acarbose, miglitol, and voglibose, are suitable inhibitors of α-glucosidase and α-amylase enzyme activities, but these drugs are known to be associated with adverse effects (Rehana et al. 2017). Owing to these drugs’ limitations, immense interest in plant-derived sources of inhibitors with minimal side-effects is currently being explored (Rehana et al. 2017). Bioactive compounds isolated from plants have proven to be potent hypoglycemic agents that can be used in drug development and treatment options for ailments (Kifle and Enyew 2020). Additionally, green-synthesized nanoparticles from metals, such as gold, iron, silver, zinc, and their oxides, have been reported for their medical and biological applications (Alkaladi et al. 2014; Gadoa et al. 2022).

The findings from the present study showed that ZnO-NPs effectively inhibited higher α-glucosidase at lower concentrations. Meanwhile, at higher concentrations, the rate of inhibitory activity of ZnO-NPs did not progress enough compared to acarbose (Fig. 14a). The ZnO-NPs showed a strong inhibitory effect against α-amylase; the highest impact with a substantial difference was at the lower concentration (70%) (Fig. 14b). The inhibitory activity of ZnO-NPs against α-glucosidase and α-amylase could be due to phytochemical compounds capping the nanoparticles (Kifle and Enyew 2020). Compounds such as polyphenols and flavonoids isolated from plants are well known for inhibiting alpha-glucosidase and alpha-amylase (Kifle and Enyew 2020). In addition, the ZnO-NPs showed potent lipase inhibition at higher concentrations. Pancreatic lipase is an enzyme responsible for converting triglyceride into fatty acid and glycerol (Ashraf et al. 2023). Several complications, such as DM, cardiovascular diseases, and hypertension, are associated with obesity caused by high levels of fat in the body (Ashraf et al. 2023). To avoid these complications, it is necessary to slow down the rate of enzyme activity involved in breaking down triglyceride into fatty acids and glycerol. Drugs such as Orlistat are commonly used to inhibit pancreatic lipase activity in obese cases. Recently, other sources of inhibitors have been reported to come from plants. Compounds, such as flavonoids, phenolics, tannins, and saponins, suppress enzymatic activity (Ashraf et al. 2023). The current finding shows that ZnO-NPs have a strong inhibitory effect on lipase activity (Fig. 13). It was observed that ZnO-NPs at higher concentrations demonstrated good inhibition compared to the standard drug, orlistat. Similarly, Meer et al. (2022) and Ashraf et al. (2023) reported better lipase inhibition of ZnO-NPs derived from *L. sativum* and *Boerhavia diffusa* Linn. seeds, respectively.

In the current study, ZnO-NPs showed a remarkable glucose uptake and glucose utilization in the treated cells when compared to the control Figs. 11a and 12a–c), suggesting an excellent antidiabetic agent for potential drug development (Asani et al. 2017; Virgen-Ortiz et al. 2020). Other in vitro studies showed that ZnO-NPs could potentially improve glucose transporter (GLUT-4) and increase β-cell proliferation (Asani et al. 2017; Virgen-Ortiz et al. 2020), by maintaining stable glucose metabolism. Glucose transporters (GLUTs) play a major role by regulating tissue-specific glucose uptake in organs, such as adipose tissue, liver, and skeletal muscles, ensuring the blood glucose level is well regulated (Chadt and Al-Hasani 2020). A combined action of GLUT-2 and GLUT-4 transporters facilitates glucose clearance in the bloodstream. GLUT-2 increases insulin secretion and binding to its receptors, helping to increase the level of GLUT-4 in the plasma membrane and inhibition of hepatic gluconeogenesis, thus promoting glucose transportation to storage organs (Wang et al. 2012; Chadt and Al-Hasani 2020).

The ability of ZnO-NPs to reduce nitrite (NO) concentration in response to LPS activation of RAW macrophages was used to determine the anti-inflammatory activities. A substantial decrease in NO levels was observed at all concentrations, with cytotoxicity also noted at these concentrations (Fig. 10a–d); therefore, the results need to be interpreted with caution. Other previous studies have reported that ZnO-NPs could reduce allergic inflammatory responses (Kim and Jeong 2015). Generally, macrophages are known to be involved in the initiation, maintenance, and resolution of inflammatory reactions within the immune systems (Watanabe et al. 2019). Activation of these macrophages through lipopolysaccharide (LPS) binding to TLR4 activates the release of inflammatory mediators such as interleukin (IL)-1 β and NO. Nitrites are known to play a fundamental role as mediators in cellular communications. However, when produced in excess, it can result in complications like neurological disorders, septic shock, rheumatoid arthritis, and autoimmune disease (Kim and Jeong 2015).

The cellular antioxidant activity of green-synthesized ZnO-NPs was determined in C3A cells using TBHP as an oxidant and CellROX^®^ Orange as a quantitative indicator of ROS. Catechin (100 μM) was used as a positive control to indicate antioxidant activity, as shown in Fig. 9A and B. It was noted that ZnO-NPs did not induce CAA activity at lower concentrations, but there was a significant increase at the highest concentration, which was also cytotoxic. The CAA activity at this concentration could not be inferred because of toxicity. The toxic effect and increased ROS production shown by the ZnO-NPs could be linked to insufficient bioactive reducing metabolites in suppressing the production of ROS (Nagajyothi et al. 2015). Other studies have shown that phytochemical capping of ZnO-NPs could suppress ROS production and exhibit minimal toxicity, contrary to observations from our current study (Liu et al. 2010; Wu et al. 2014; Nagajyothi et al. 2015). Also, the elevated ROS generation might be due to damage to the mitochondrial electron transport chain, which then alters protein activity and subsequent cell death or apoptosis (Bhattacharya et al. 2020; Zhang et al. 2023). Based on our findings, it could be assumed that the lower concentration of ZnO-NP (Fig. 9) exhibited good CAA activity, whereas, at the highest concentration, there was toxicity and cell death due to ROS generation due to mitochondrial damage and oxidative stress (Choudhury et al. 2017; Wang et al. 2018; Zhang et al. 2023).

Similarly, the previous studies have reported increased ROS generation following treatment with increased concentrations of ZnO-NPs (Choudhury et al. 2017). The generation of ROS has been attributed to the release of excessive intracellular zinc ions; thus, treatment with ZnO-NPs could trigger a singlet oxygen state (^1^O_2_) (Choudhury et al. 2017; Lekki-Porębski et al. 2023). Based on these reports, it is plausible to suggest that ZnO-NPs are toxic with increasing concentration (Bandeira et al. 2020. This finding corroborates the other findings that have reported the cytotoxic effects of ZnO-NPs on many different cells (Anitha et al. 2018; Mkhize et al. 2022).

## Conclusion

The current study synthesized and characterized ZnO-NPs of *H. cymosum* aqueous extract. The ZnO-NPs were further assessed for antidiabetic, anti-inflammatory, cellular antioxidant, and cytotoxicity potentials. Notably, the biological activities of ZnO-NPs were enhanced by the presence of the phytochemical compounds acting as the capping agents. The excellent inhibitory activity of α-glucosidase, α-amylase, lipase inhibition glucose uptake, and utilization demonstrated by ZnO-NPs make it a promising candidate that could be optimized further and used as a lead in the production of antidiabetic drugs. The anti-inflammatory results could not be adequately interpreted because of the observation of toxicity. Further in vitro and in vivo antidiabetic studies of ZnO-NP are recommended. This study is the first to explore the antidiabetic potential of ZnO-NPs synthesized using aqueous extract of *H. cymosum*.

## Data Availability

The datasets used and/or analyzed during the current study are available from the corresponding author on reasonable request.
